# Transcranial Current Stimulation Alters the Expression of Immune-Mediating Genes

**DOI:** 10.3389/fncel.2019.00461

**Published:** 2019-10-25

**Authors:** Monika Rabenstein, Marcus Unverricht-Yeboah, Meike Hedwig Keuters, Anton Pikhovych, Joerg Hucklenbroich, Sabine Ulrike Vay, Stefan Blaschke, Anne Ladwig, Helene Luise Walter, Magdalena Beiderbeck, Gereon Rudolf Fink, Michael Schroeter, Ralf Kriehuber, Maria Adele Rueger

**Affiliations:** ^1^Department of Neurology, University Hospital of Cologne, Cologne, Germany; ^2^Radiation Biology Unit, Department of Safety and Radiation Protection, Research Centre Jülich, Jülich, Germany; ^3^Max Planck Institute for Metabolism Research, Cologne, Germany; ^4^A.I. Virtanen Institute for Molecular Sciences, University of Eastern Finland, Kuopio, Finland; ^5^Cognitive Neuroscience, Institute of Neuroscience and Medicine (INM-3), Research Centre Jülich, Jülich, Germany

**Keywords:** direct transcranial current stimulation, gene expression, microarray, MHC-I, osteopontin, transcriptome

## Abstract

Despite its extensive use in clinical studies, the molecular mechanisms underlying the effects of transcranial direct current stimulation (tDCS) remain to be elucidated. We previously described subacute effects of tDCS on immune- and stem cells in the rat brain. To investigate the more immediate effects of tDCS regulating those cellular responses, we treated rats with a single session of either anodal or cathodal tDCS, and analyzed the gene expression by microarray; sham-stimulated rats served as control. Anodal tDCS increased expression of several genes coding for the major histocompatibility complex I (MHC I), while cathodal tDCS increased the expression of the immunoregulatory protein osteopontin (OPN). We confirmed the effects of gene upregulation by immunohistochemistry at the protein level. Thus, our data show a novel mechanism for the actions of tDCS on immune- and inflammatory processes, providing a target for future therapeutic studies.

## Introduction

Transcranial direct current stimulation (tDCS) has been applied in experimental and clinical settings for more than 20 years and may facilitate rehabilitation after stroke as suggested by clinical data (Hummel et al., 2005; Sparing et al., 2009). Moreover, tDCS is used as experimental therapy for various neurological and psychiatrical diseases, e.g., multiple sclerosis, Parkinson’s, depression, dementia, evaluated by Lefaucheur et al. (2017). TDCS leads to changes of the cortical excitability in animals and humans (Bindman et al., 1962; Nitsche and Paulus, 2000), promoting changes in long-term potentiation and synaptic plasticity via NMDA-receptors (Bolin et al., 2002; Nitsche et al., 2003; Fritsch et al., 2010; Monte-Silva et al., 2013; Kronberg et al., 2017). As tDCS can be applied either using anodal or a cathodal current polarity, anodal tDCS increases cortical excitability while cathodal tDCS results in its decrease (Fritsch et al., 2010; Stagg and Nitsche, 2011; Lafon et al., 2017).

Intriguingly, tDCS evokes various cellular effects on neural stem cells, neurons, astrocytes, oligodendrocytes, and microglia exceeding its primary neurophysiological actions: In the healthy rat brain, tDCS increases proliferation and migration of endogenous neural stem cells and activates microglia as the brain-resident immune cells (Rueger et al., 2012a; Keuters et al., 2015). Both anodal and cathodal tDCS induce neurogenesis, both in healthy animals (Braun et al., 2016) as well as after experimental stroke (Braun et al., 2016; Pikhovych et al., 2016). Cathodal tDCS recruits oligodendrocyte precursors toward an ischemic lesion while supporting polarization of microglia toward a pro-inflammatory M1-phenotype (Braun et al., 2016). Depending on the current density, tDCS downregulates inflammatory mediators (Peruzzotti-Jametti et al., 2013) and the constitutive expression of ionized calcium binding adaptor molecule 1 (Iba1) by activated microglia (Spezia Adachi et al., 2012; Peruzzotti-Jametti et al., 2013; Pikhovych et al., 2016). These data suggest that – depending on current density and polarity – tDCS possesses distinct immunomodulatory effects and supports stem cell-mediated regeneration in the brain. Further knowledge of the patterns of action of the different polarities is still warranted. Cellular effects of tDCS occur from acute effects within hours to subacute and longterm effects within days and weeks after stimulation (Rueger et al., 2012b; Spezia Adachi et al., 2012; Peruzzotti-Jametti et al., 2013; Braun et al., 2016; Pikhovych et al., 2016). It is yet unknown how they are regulated and altered over time. Expression changes on the transcriptome can be expected after a few hours after a stimulus, therefore we chose to investigate the acute tDCS-induced transcriptome in an unbiased microarray approach 6 h after tDCS.

## Materials and Methods

### Animals and Surgery

All animal procedures followed the German Laws for Animal Protection and were approved by the local animal care committee as well as local governmental authorities (Landesamt für Natur, Umwelt und Verbraucherschutz North Rhine-Westphalia, LANUV). To exclude putative influences of hormonal changes on the findings, only male rats were used.

Spontaneously breathing 10–11 weeks old male Wistar rats weighing 260–310 g were anesthetized with 5% isoflurane and maintained with 2.5% isoflurane in 65%/35% nitrous oxide/oxygen. Throughout surgical procedures, body temperature was maintained at 37.0°C with a thermostatically controlled heating pad.

### Transcranial Direct Current Stimulation

Twenty-four rats were subjected to a single tDCS session as described previously by our group (Rueger et al., 2012a; Braun et al., 2016). In brief, an epicranial electrode holder made of plastic (self-manufactured) with a defined contact area of 3.5 mm^2^ was mounted onto the intact skull over the right hemisphere using non-toxic glass ionomer luting cement (Ketac Cem Plus, 3M-ESPE, Germany) at bregma AP + 2.0 mm, ML + 2.0 mm, and left in place for the entire experiment. The skin around the electrode holder was closed with sutures after the placement. The holder was left in place for the entire experiment. Animals were randomized to receive tDCS with either anodal (*n* = 8) or cathodal (*n* = 8) polarity; the control group received a sham-stimulation (*n* = 8) (Table 1).

**TABLE 1:** Overview of the experimental groups.

	**Microarray**	**Immunohistochemistry**
Sham	*n* = 4	*n* = 4
Anodal	*n* = 4	*n* = 4
Cathodal	*n* = 4	*n* = 4

For transcranial direct current stimulation, an argentic electrode was placed in the electrode holder, and 0.9% sodium chloride was added to buffer electrochemical changes. The counter electrode, a 1.5 cm × 2 cm silver-coated sensor electrode (#DENIS01526; Spes Medica, Genova, Italy), was placed on the rat’s ventral thorax.

Transcranial direct current stimulation was applied continuously for 15 min at 500 μA using a constant current stimulator (CX-6650, Schneider-Electronics, Germany) under isoflurane anesthesia, resulting in a charge density of 128 kC/m2. Charge density was calculated as charge (A × s) per area, according to Liebetanz et al., 2009. For sham stimulation, rats were treated equally to the tDCS group with isoflurane anesthesia for 15 min, but were not connected to the current stimulator during this time. tDCS was performed under anesthesia to avoid dislocation of the cable.

After tDCS, animals were allowed to recover in their home cages with access to food and water *ad libitum*.

### RNA-Extraction

Six hours after tDCS, four rats of each stimulation group were deeply anesthetized and decapitated. The brains were rapidly removed and the sensorimotor cortices of each hemisphere were isolated. 20mg cortical tissue of each hemisphere was crushed and stabilized overnight in PurifyLater Tissue Stabilizer (BioEcho, Dormagen, Germany). On the next day, the total RNA was isolated using the GenUP^TM^ Total RNA Kit (Biotechrabbit, Henningsdorf, Germany) according to the manufacturer’s guidelines. RNA quantification was carried out using a NanoDrop-1000 spectrophotometer (Peqlab, Erlangen, Germany), and RNA quality was monitored by agarose gel separation and with the Agilent 2100 Bioanalyzer (Agilent, Böblingen, Germany). All extracted RNA samples were found to be of good quality. RNA integrity numbers (RINs) ranged from 9.8 to 10.

### DNA Microarray Hybridization

DNA microarray experiments were performed according to the manufacturer’s manual and as previously described (Unverricht-Yeboah et al., 2018). Of the total RNA, 400 ng was transcribed into cDNA with an oligo-dT primer, followed by transcription into cRNA labeled with cyanine 3-CTP (Quick-Amp Labeling Kit, One-color, Agilent, Santa Clara, CA, United States). cRNA purification was performed with the RNeasy Mini Kit (Qiagen). cRNA yield and the dye incorporation were measured with the NanoDrop-1000 spectrophotometer. Labeled cRNA samples were hybridized for 17 h to 44 k Whole Rat Genome DNA microarrays (G2519F, Agilent) using a hybridization oven (Agilent). After hybridization and washing, DNA microarrays were scanned with the Microarray Scanner (G2505 B, Agilent) as recommended by Agilent.

### Data Analysis

Images of the scanned microarrays were processed with the Agilent Feature Extraction software. Gene expression data were processed, normalized, and analyzed using the GeneSpring GX software (Agilent) and Excel (Microsoft Corperation, Redmond, WA, United States). By data filtering, non-uniform outliers were excluded, as well as signals that were not significantly above the background intensity in at least 25% of the samples. To indicate the significantly regulated genes, the *p*-values were adjusted using the method of Benjamini and Hochberg to calculate the false discovery rate (FDR). Genes with non-FDR adjusted *p*-values were considered for the filtering process to increase the number of potential candidate genes. The criteria for candidate genes were: a significant expression change (>2.0 fold), a FDR ≤ 0.16 after filtering, and a *p*-value < 0.05 (Table 2). Protein expression of selected candidate genes was analyzed by immunohistochemistry.

**TABLE 2:** Numbers of significantly regulated genes after tDCS with a fold change > 2.

**Groups**	**Significantly regulated genes (fold change > 2.0, *p* < 0.05, FDR ≤ 0.16)**
Cathodal ipsilateral vs. Sham	20
Anodal ipsilateral vs. Sham	14
Cathodal contralateral vs. Sham	0
Anodal contralateral vs. Sham	0
Cathodal ipsilateral vs. Cathodal contralateral	1
Anodal ipsilateral vs. Anodal contralateral	0

### Functional Analysis of Significantly Regulated Genes

The significantly expressed genes after anodal and cathodal tDCS were functionally categorized using the Database for Annotation Visualization and Integrated Discovery 6.8 (DAVID) (Huang et al., 2009). To assign the significantly altered genes to affected biological processes and pathways, we used the gene ontology analysis feature.

### Immunohistochemistry

Six hours after tDCS, four rats of each stimulation group were deeply anesthetized with isoflurane and decapitated. Brains were rapidly removed, frozen in isopentane, and stored at –80°C before further processing. Serial coronal brain sections of 10 μm were cut throughout each brain at 500 μm intervals. For immunohistochemistry, primary antibodies included: MHC Class I Antibody (Ox18) (1:500 cat# NB120-6405, Novus Biological, Littleton, CO, United States) and anti-Osteopontin-Antibody (1:500 cat# ab8448, Abcam, Cambridge, United Kingdom), Iba1 Antibody (1:1000 cat# 019-19741WAKO, Osaka, Japan), and NeuN Antibody (1:200, cat# MAB377 Merck, Kenilworth, NJ, United States). For visualization of the MHC I-antibody, the ABC-Elite kit (Vector Laboratories, United States) with diaminobenzidine (Sigma, Germany) as the final reaction product was used. For visualization of the other antibodies, fluorescent-labeled secondary antibodies were used (1:500, cat#A11001 Alexa-Fluor–488 and cat#A11036 Alexa-Fluor–568, Invitrogen, Thermo Fisher Scientific, Waltham, MA, United States). Sections were counterstained with Hoechst to label all nuclei (Hoechst 33,342, Thermo Fisher Scientific, Massachusetts, United States).

Representative pictures were taken with an inverted fluorescence phase-contrast microscope (Keyence BZ-9000E, Keyence, Osaka, Japan).

To determine the amount of MHC Class I-positive cells, the size of the subventricular zone (SVZ) was quantified for each animal by measuring the area in μm^2^ covered by MHC Class I-positive cells with a predefined length at 100 μm multiplied with the width in μm of the area covered with MHC + cells [as previously described by our group for doublecortin stainings (Klein et al., 2014)], and calculated as the mean of three coronal sections at 500 μm intervals of each animal.

To determine the number of OPN-positive, NeuN-positive, and Iba1-positive cells in the cortex, three coronal sections at 500 μm intervals were stained with the respective antibody. Using a Keyence microscope with a 40× objective, images of representative fields of view (measuring 746 μm × 557 μm) of the sensorimotor cortex of the ipsilateral, stimulated hemisphere were taken of each section. The numbers of positive cells of each antibody staining were counted manually and calculated as the mean of three coronal sections.

Immunohistochemical quantifications of MHC Class I and OPN positive cells were performed by a blinded evaluator (MS).

Descriptive statistics were performed with Graph Pad Prism (GraphPad Software Inc.). For comparison of multiple groups, One Way Analysis of Variance (ANOVA) and Tukey’s *post hoc* tests were performed with the same software. Statistical significance was set at the <5% level (*p* < 0.05).

## Results

### Gene Expression Changes Following Different tDCS Polarities

Six hours after cathodal ipsilateral tDCS (compared to sham stimulation), 20 genes were significantly up- or downregulated (10 genes up-, 10 genes downregulated, cmp. Supplementary Table S1A). After anodal ipsilateral tDCS (compared to sham stimulation), 14 genes were significantly up- or downregulated (9 genes up-, 5 genes downregulated, cmp. Supplementary Table S1B). Of all the other groups, comparing ipsilaterally stimulated to contralaterally stimulated hemispheres, or contralaterally stimulated hemispheres to sham stimulation, only cathodal ipsilateral tDCS compared to cathodal contralateral stimulation resulted in a significant difference of one gene that was downregulated (Supplementary Table S1C).

The effects of tDCS were lateralized in comparison to sham but not in comparison to the unstimulated hemisphere. Given the small size of a rat brain, tDCS stimulation was not assumed to be restricted to one hemisphere. Therefore, tDCS effects will also reflect in the unstimulated hemisphere, albeit to a lower extent, which explains the lack of significant changes between the unstimulated hemisphere and sham. Thus, the “unstimulated” hemisphere cannot function as control. We therefore used sham control animals, specifically from the same hemisphere that was stimulated in the tDCS group.

### Biological Processes and Pathways Affected by Different tDCS Polarities

We functionally categorized the upregulated genes, using DAVID (Huang et al., 2009), to examine biological processes and pathways affected by the different tDCS polarities (Supplementary Figure S1). Six hours after anodal tDCS, the categories “antigen presentation via MHC I” and “immune response” were significantly upregulated, both consisting of the same 5 different genes coding for RT1 Class (MHC I), thus strongly suggesting an upregulation of MHC I coding genes after anodal tDCS (Supplementary Figure S1A).

After cathodal tDCS, the categories “osteoblast differentiation,” “positive regulation of angiogenesis,” “cellular response to mechanical stimuli,” “ossification,” and “response to activity” were significantly upregulated (Supplementary Figure S1B). For the downregulated genes in cathodal and anodal tDCS no functional categories could be detected.

### Identification of Candidate Genes for Immunomodulation

**FIGURE 1:**
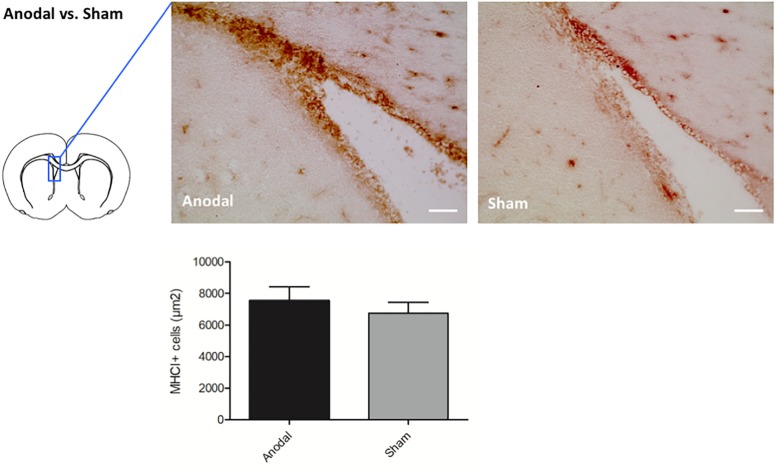
Effects of anodal tDCS on protein expression. Representative immunohistochemical images of Ox18 + cells in the SVZ ipsilateral to anodal tDCS or sham stimulation. Staining for Ox18 (MHC I) in the ipsilateral SVZ revealed more Ox18 + cells in animals treated by anodal tDCS (left panel) compared to sham stimulation (right panel) by trend (scale bars = 100 μm). Results are displayed as mean ± SEM.

To confirm the effects of upregulated genes seen in the microarray analysis by immunohistochemistry, we selected candidate genes for each stimulation polarity. Given the strong effects of tDCS on neuroinflammation found in earlier studies of our group (Rueger et al., 2012a; Braun et al., 2016; Pikhovych et al., 2016), we focused on genes coding for immunomodulating processes. After anodal tDCS, we identified a cluster of 5 upregulated genes coding for RT1 Class as part of the MHC I complex (RT1-CE2, RT1-CE15, RT1-CE16, Rt1.aa, and RT1-EC2) that we singled out as candidate genes. We performed immunohistochemical stainings for MHC I on brain slices from animals that had been identically treated as the microarray group. Quantification showed more MHC I + cells in the ipsilateral subventricular zone (SVZ) of animals treated with anodal tDCS compared to controls by trend (anodal tDCS 7533,75 ± 881,41 MHC I + cells/μm^2^ vs. sham stimulation 6758,33 ± 675,57 MHC I + cells/μm^2^). Results are displayed as mean ± SEM (Figure 1).

**FIGURE 2:**
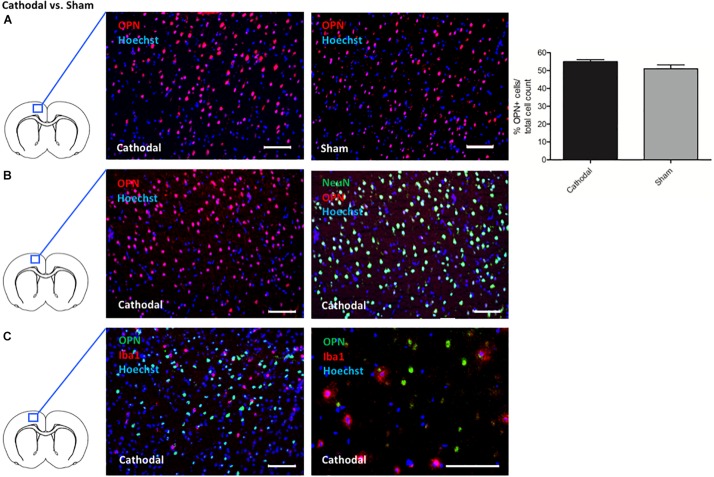
Effects of cathodal tDCS on protein expression. **(A)** Representative immunohistochemical images of OPN + cells (red) in the cortex ipsilateral to cathodal tDCS or sham stimulation, co-stained with a nuclear marker (Hoechst; blue). Staining for OPN revealed more OPN + cells in the cortex of animals treated by cathodal tDCS (left panel) compared to sham stimulation (right panel) by trend (right panel; scale bars = 100 μm). Results are displayed as mean ± SEM. **(B)** Representative immunohistochemical images of OPN + cells co-stained with NeuN (green) in the cortex ipsilateral to cathodal tDCS. All images were co-stained with a cell nucleus marker/DNA dye (Hoechst; blue). OPN + cells almost exclusively co-expressed NeuN (left panel and right panel represent the same picture with and without NeuN fluorescence signal, scale bars = 100 μm). **(C)** Representative immunohistochemical images of OPN + cells (green) co-stained with Iba1 (red) in the cortex ipsilateral to cathodal tDCS. All images were co-stained with a cell nucleus marker/DNA dye (Hoechst; blue). OPN + cells did not express Iba1 (left panel and right panel represent different magnifications, scale bars = 100 μm).

Intriguingly, cathodal tDCS led to upregulation of Spp1 encoding for the phosphoprotein osteopontin (OPN), possessing pleiotropic immunoregulatory properties after cerebral ischemia as well as beneficial effects on endogenous neural stem cells (Rabenstein et al., 2015, 2016; Ladwig et al., 2017). To validate the involvement of OPN, we performed immunohistochemical stainings in animals that had been identically treated as the microarray group. Quantification showed more OPN + cells in ipsilateral the cortex of animals treated with cathodal tDCS compared to sham-treated controls by trend (cathodal tDCS 54.89 ± 1.29% OPN + cells vs. sham stimulation 51.02 ± 2.16% OPN + cells/total cell count). Results are displayed as mean ± SEM (Figure 2A). Co-staining OPN with either NeuN for neurons or Iba1 for microglia revealed that OPN was almost exclusively expressed by neurons (Figures 2B,C).

### Other Genes of Interest

In cathodal tDCS, the Slitrk6 inhibitor of neurite outgrowth was significantly downregulated (Aruga and Mikoshiba, 2003). While bone morphogenic protein 6 (bmp-6), a secreted extracellular matrix (ECM)-associated component with important functions in development, was upregulated, its antagonist Sostdc1 was downregulated (Brazil et al., 2015). Sostdc 1 was also significantly downregulated after anodal tCDS, but without upregulation of bone morphogenic protein.

Both cathodal and anodal tDCS upregulated Wisp2 (Ohkawa et al., 2011) that leads to the facilation of neurite formation. Wisp2 is part of the Wnt signaling pathway involved in neural stem cell development (Bengoa-Vergniory and Kypta, 2015).

Together, both in cathodal and anodal tDCS, neurite growth and stem cell development were enhanced, while extracellular developmental processes were enhanced after cathodal tDCS only.

## Discussion

A single session of tDCS led to significant changes in gene expression after 6 h, which were accompanied by concordant changes at the protein level as detected immunohistochemically. The rationale behind this short interval after tDCS was to observe the acute of effects tDCS within the first hours.

Changes in protein upregulation were not statistically significant, most likely since their expression maximum occurred at later time points (Jansen and Pfaffelhuber, 2015). Moreover, increased protein synthesis may not necessarily lead to an increased number of positive cells, but to an increased amount of gene product per cell. Further studies are warranted to address the issue of induced protein expression.

After anodal tDCS, five genes involved in MHC I expression were upregulated. In the healthy brain, MHC-I is expressed at the synapses of neurons (Shatz, 2009; Needleman et al., 2010). It is generally associated with negative regulation of neural plasticity (Datwani et al., 2009; Shatz, 2009; Elmer and McAllister, 2012); on the other hand, decreased MHC I signaling impairs axonal repair (Thams et al., 2008). After brain damage such as stroke, MHC-I is upregulated, and MHC-I presenting cells are recognized by cytotoxic T-cells, supporting neuroinflammation (Schroeter et al., 1994; Piehl and Lidman, 2001). Interestingly, MHC-I is upregulated after kainate-induced seizure and downregulated after activity-blockage (Corriveau et al., 1998). Thus, upregulation of genes coding for MHC-I by anodal tDCS – potentially inducing cortical excitability – is in line with previous reports linking altered neuronal activity directly to MHC I expression (Neumann et al., 1995, 1997). Data suggest that the increase in neuroinflammation seen after tDCS (Rueger et al., 2012a; Braun et al., 2016; Pikhovych et al., 2016) may result from an upregulation of MHC-I that tags neurons to surveillance of microglia, the immunocompetent cells of the CNS. Together, anodal tDCS may act by upregulating MHC I expression through an increase in cortical excitability, leading to an augmented neuroinflammatory response.

Cathodal tDCS led to an upregulation of OPN as an endogenous phosphoglycoprotein with essential roles in tissue homeostasis, wound healing, immune regulation, and stress responses (Denhardt et al., 2001; Brown, 2012). OPN acts as a negative feedback regulator for the synthesis of nitric oxide (Hwang et al., 1994; Rollo et al., 1996), suggesting key immunoregulatory functions. OPN increases survival, proliferation, migration, and neuronal differentiation of endogenous neural stem cells in culture, and enhances proliferation and migration of neuronal precursors *in vivo* after cerebral ischemia (Rabenstein et al., 2015). Additionally, OPN seems to polarize microglia to a neuroprotective subtype in an inflammation setting (Rabenstein et al., 2016). Thus, upregulating OPN by cathodal tDCS may provide an easily accessible non-pharmacological approach to enhance OPN synthesis in order to harness its beneficial effects, e.g., after a stroke. In conclusion, we speculate that cathodal tDCS leads to a multitude of regulatory neuroinflammatory and neuroplastic effects through upregulation of OPN.

As yet, only Holmes et al. conducted an RNA-sequencing study after tDCS (Holmes et al., 2016). They analyzed gene expression only minutes after a single session of anodal tDCS using three different currents (250, 500, and 2,000 μA), equivalent to charge densities of 132, 264, and 1,057 kC/m^2^), while our study was performed 6 h after tDCS of either anodal or cathodal polarity at 128 kC/m^2^. For each charge density, Holmes et al. found about 1000 genes differentially up- and downregulated, especially from inflammatory, antidepressant-related, and receptor signaling pathways. In contrast to our study, they chose a fold change of 1.2 with adjusted *p*-value < 0.1 as the cut-off for significantly regulated genes, while we used a more conservative approach with only >2-fold changes, explaining the larger number of differentially regulated genes in their study. At 132 kC/m^2^, similar to the charge density in our study, cellular response to stress- and B-cell activation-clusters, and only at 1,057 kC/m^2^ – far above the published lesion threshold (Liebetanz et al., 2009; Rueger et al., 2012a) - a favorable adjustment of immune system regulation clusters was found.

As previously suggested by Liebetanz et al. (2009), another recent study found that subtle lesions can possibly occur even below the current intensity used in our study (Jackson et al., 2017). While this certainly needs to be kept in mind for future studies, we here did not detect any tissue lesions by immunohistochemistry using the current stimulation parameters.

Further studies are warranted to establish the effects of tDCS on gene expression not only in relation to timing but also to charge density and polarity, as well as in awake stimulated animals.

## Conclusion

Anodal tDCS enhanced expression of several genes coding for MHC-I, affecting inflammation and synaptic plasticity, while cathodal tDCS increased expression of the gene encoding for the immunoregulatory protein OPN linking tDCS treatment to beneficial effects on regeneration after stroke or cerebral hemorrhage (Yan et al., 2009; Wu et al., 2011; Rabenstein et al., 2015; Ladwig et al., 2017; Rogall et al., 2018). Overall, specific modulation of neuroinflammatory processes by non-invasive brain stimulation constitutes a promising therapeutic option with immediate translational relevance.

## Data Availability Statement

Microarray data can be found on ArrayExpress (accessionE-MTAB-8318).

## Ethics Statement

The animal study was reviewed and approved by the Landesamt für Natur, Umwelt und Verbraucherschutz North Rhine-Westphalia.

## Author Contributions

MR performed the microarray analyses, gene ontology analyses, and immunohistochemical stainings, and drafted the manuscript. AP and MK carried out the surgery. SV, MB, SB, AL, and HW helped with the immunohistochemical stainings. MR and JH performed the RNA isolation. MU-Y performed the microarray and helped with statistical analyses. GF, RK, and MS participated in the design and coordination of the study, and helped to draft and critically revised the manuscript. MAR conceived of, designed, and coordinated the study, helped with the statistical analyses, and finalized the manuscript.

## Conflict of Interest

The authors declare that the research was conducted in the absence of any commercial or financial relationships that could be construed as a potential conflict of interest.

## Supplementary Material

FIGURE S1 Effects of tDCS on gene regulation. Genes significantly expressed 6 h after anodal tDCS (A) respectively, cathodal tDCS (B) were functionally categorized. Affected biological processes and pathways as well as the respective enrichment p-values are shown.

TABLE S1 Significantly up- and downregulated genes after tDCS.

## Abbreviations

DAVID: database for annotation visualization and integrated discovery 6.8
FDR: false discovery rate
Iba1: ionized calcium binding adaptor molecule 1
MHC I: major histocompatibility complex class I
OPN: osteopontin
tDCS: transcranial direct current stimulation
SVZ: subventricular zone.
