# Supplementary material for: Transcranial Current Stimulation Alters the Expression of Immune-Mediating Genes
Source: Front Cell Neurosci. 2019 Oct 25;13:461. doi: 10.3389/fncel.2019.00461 (PMC6824260; doi:10.3389/fncel.2019.00461)
Supplement: FIGURE S1 — Effects of tDCS on gene regulation. Genes significantly expressed 6 h after anodal tDCS (A) respectively, cathodal tDCS (B) were functionally categorized. Affected biological processes and pathways as well as the respective enrichment p-values are shown. [file Data_Sheet_1.PDF]

A. Significantly up- and downregulated genes after cathodal tDCS of the ipsilateral cortex compared to sham stimulation					
**Probe Name**	**Gene Symbol**	**Description**	**FC**	**P-Value**	**P-Value (FDR)**
A_42_P471653	Anxa8	ref|Rattus norvegicus annexin A8 (Anxa8), mRNA [NM_001031654]	2,60	0,0004	0,055607
A_44_P299247	Aqp1	ref|Rattus norvegicus aquaporin 1 (Aqp1), mRNA [NM_012778]	2,16	0,0060	0,140538
A_42_P575104	Sostdc1	ref|Rattus norvegicus sclerostin domain containing 1 (Sostdc1), mRNA [NM_153737]	0,42	0,0062	0,140538
A_44_P491796	Spp1	ref|Rattus norvegicus secreted phosphoprotein 1 (Spp1), mRNA [NM_012881]	2,12	0,0107	0,140538
A_43_P15980	Angpt2	ref|Rattus norvegicus angiopoietin 2 (Angpt2), mRNA [NM_134454]	2,18	0,0114	0,140538
A_42_P629321	Wisp2	ref|Rattus norvegicus WNT1 inducible signaling pathway protein 2 (Wisp2), mRNA [NM_031590]	2,41	0,0142	0,140538
A_44_P314110	Ermn	ref|Rattus norvegicus ermin, ERM-like protein (Ermn), mRNA [NM_001008311]	0,44	0,0190	0,140538
A_44_P639365	TC606727	tc|Q569E3_MOUSE (Q569E3) Col24a1 protein, partial (35%) [TC606727]	0,42	0,0268	0,140538
A_44_P884935	Plekhh1	ref|Rattus norvegicus pleckstrin homology domain containing, family H (with MyTH4 domain) member 1 (Plekhh1), mRNA [NM_001108036]	0,50	0,0270	0,140538
A_64_P161216	Kif5c	ref|Rattus norvegicus kinesin family member 5C (Kif5c), mRNA [NM_001107730]	2,43	0,0289	0,140538
A_44_P314087	LOC681186	ref|PREDICTED: Rattus norvegicus hypothetical protein LOC681186 (LOC681186), mRNA [XM_001060674]	2,18	0,0316	0,140538
A_64_P051932	Mdh1b	ref|Rattus norvegicus malate dehydrogenase 1B, NAD (soluble) (Mdh1b), mRNA [NM_001108221]	0,42	0,0317	0,140538

Probe Name	Gene Symbol	Description	FC	P-Value	P-Value (FDR)
A_42_P740370	Cpxm2	ref|Rattus norvegicus carboxypeptidase X (M14 family), member 2 (Cpxm2), mRNA [NM_001106306]	2,44	0,0325	0,140538
A_44_P250030	Bmp6	ref|Rattus norvegicus bone morphogenetic protein 6 (Bmp6), mRNA [NM_013107]	2,31	0,0327	0,140538
A_42_P594228	Abca8a	ref|Rattus norvegicus ATP-binding cassette, subfamily A (ABC1), member 8a (Abca8a), mRNA [NM_001281824]	0,49	0,0395	0,140538
A_43_P13102	Ada	ref|Rattus norvegicus adenosine deaminase (Ada), mRNA [NM_130399]	2,44	0,0396	0,140538
A_64_P022412	Anln	ref|PREDICTED: Rattus norvegicus similar to Anillin (RGD1561367), mRNA [XM_001077789]	0,49	0,0402	0,140538
A_64_P129662	Slitrk6	ref|Rattus norvegicus SLIT and NTRK-like family, member 6 (Slitrk6), mRNA [NM_001106057]	0,43	0,0454	0,140538
A_64_P012373	Akr1c13	ref|Rattus norvegicus aldo-keto reductase family 1, member C13 (Akr1c13), mRNA [NM_001014240]	0,39	0,0484	0,144527
A_64_P116299	XM_343725	gb|Rattus norvegicus similar to RIKEN	0,48	0,0492	0,144527

**B. Significantly up- and downregulated genes after anodal tDCS of the ipsilateral cortex compared to sham stimulation**					
**Probe Name**	**Gene Symbol**	**Description**	**FC**	**P-Value**	**P-Value (FDR)**
A_43_P23362	Ttc8	ref|Rattus norvegicus tetratricopeptide repeat domain 8 (Ttc8), mRNA [NM_001106752]	2,70	0,0026	0,157678
A_42_P575104	Sostdc1	ref|Rattus norvegicus sclerostin domain containing 1 (Sostdc1), mRNA [NM_153737]	0,32	0,0036	0,157678
A_64_P141747	RGD1311874	ref|Rattus norvegicus hypothetical LOC300751 (RGD1311874), mRNA [NM_001106825]	4,24	0,0064	0,157678
A_44_P107372	RT1-CE2	ref|Rattus norvegicus RT1 class I, locus CE2 (RT1-CE2), mRNA [NM_001008840]	2,26	0,0073	0,157678
A_64_P100963	Ncaph	ref|PREDICTED: Rattus norvegicus non-SMC condensin I complex, subunit H (Ncaph), misc_RNA [XR_085785]	0,41	0,0111	0,157678
A_64_P132069	RT1-CE15	ref|Rattus norvegicus RT1 class I, locus CE15 (RT1-CE15), mRNA [NM_001008838]	2,13	0,0117	0,157678
A_42_P695401	Ccl2	ref|Rattus norvegicus chemokine (C-C motif) ligand 2 (Ccl2), mRNA [NM_031530]	3,28	0,0120	0,157678
A_64_P108389	Plekhh1	ref|Rattus norvegicus pleckstrin homology domain containing, family H (with MyTH4 domain) member 1 (Plekhh1), mRNA [NM_001108036]	0,33	0,0159	0,157678
A_42_P629321	Wisp2	ref|Rattus norvegicus WNT1 inducible signaling pathway protein 2 (Wisp2), mRNA [NM_031590]	2,81	0,0164	0,157678
A_64_P068027	TC598787	gb|Rattus norvegicus similar to RIKEN cDNA 4933431D05 (LOC302278), mRNA [XM_217557]	0,46	0,0171	0,157678
A_42_P594228	Abca8a	ref|Rattus norvegicus ATP-binding cassette, subfamily A (ABC1), member 8a (Abca8a), mRNA [NM_001281824]	0,45	0,0185	0,157678
A_44_P867246	RT1-CE16	ref|Rattus norvegicus RT1 class I, locus CE16 (RT1-CE16), mRNA [NM_001008839]	3,01	0,0185	0,157678

Probe Name	Gene Symbol	Description	FC	P-Value	P-Value (FDR)
A_64_P006625	Rt1.aa	ref|Rattus norvegicus MHC class I RT1.Aa alpha-chain (Rt1.aa), mRNA [NM_001134701]	2,65	0,0197	0,157678
A_44_P324707	RT1-EC2	ref|Rattus norvegicus RT1 class Ib, locus EC2 (RT1-EC2), mRNA [NM_012645]	2,16	0,0212	0,159877

C. Significantly up- and downregulated genes after cathodal tDCS in the ipsilateral cortex compared to the contralateral cortex					
**Probe Name**	**Gene Symbol**	**Description**	**FC**	**P-Value**	**P-Value (FDR)**
A_44_P639365	TC606727	tc|Q569E3_MOUSE (Q569E3) Col24a1 protein, partial (35%) [TC606727]	0,42	0,000066	0,002054282
