# Supplementary figures and images for: Transcranial Current Stimulation Alters the Expression of Immune-Mediating Genes
Source: Front Cell Neurosci. 2019 Oct 25;13:461. doi: 10.3389/fncel.2019.00461 (PMC6824260; doi:10.3389/fncel.2019.00461)

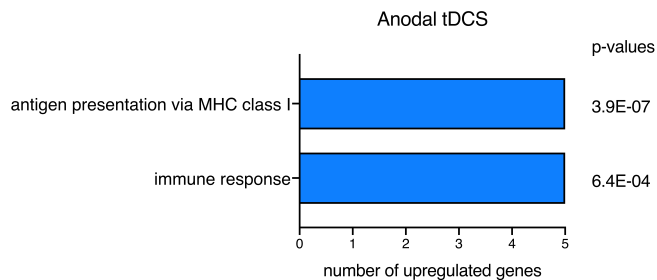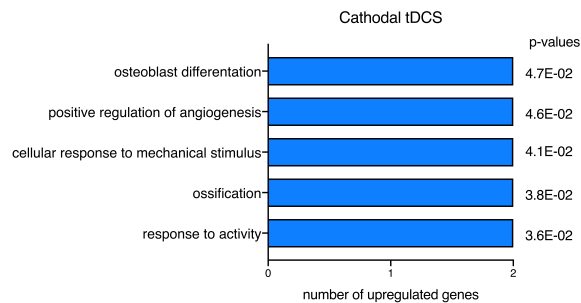

Supplement: TABLE S1 — Significantly up- and downregulated genes after tDCS. [file Data_Sheet_2.PDF]
